# Content and Quality of Information Websites About Congenital Heart Defects Following a Prenatal Diagnosis

**DOI:** 10.2196/ijmr.3819

**Published:** 2015-01-21

**Authors:** Tommy Carlsson, Gunnar Bergman, Anna-Malin Karlsson, Elisabet Mattsson

**Affiliations:** ^1^Department of Public Health and Caring SciencesUppsala UniversityUppsalaSweden; ^2^Department of Women’s and Children’s HealthKarolinska InstitutetStockholmSweden; ^3^Department of Scandinavian LanguagesUppsala UniversityUppsalaSweden; ^4^Department of Women’s and Children’s HealthUppsala UniversityUppsalaSweden

**Keywords:** consumer health information, heart defects, congenital, Internet, prenatal diagnosis

## Abstract

**Background:**

Pregnant women and their partners use the Internet to search for information following a prenatal diagnosis of congenital heart defect.

**Objective:**

Our aim was to explore central subjects of content and to assess the accessibility, reliability, usability, and quality of written information on publicly available information websites about congenital heart defects following a prenatal diagnosis.

**Methods:**

Following searches on Bing and Google, we included websites containing patient information in English. Hits ranged from 340,000-67,500,000 and the first 50 hits from each search were screened for inclusion (N=600). Of these hits, 39.3% (236/600) were irrelevant. A total of 67 websites were included, of which 37% (25/67) were affiliated with independent information websites, 25% (17/67) with charity/private organizations, 25% (17/67) with hospitals/clinics, and 13% (8/67) had other affiliations. The majority of the websites (76%, 51/67) could not be attributed to an author. A manifest content analysis was performed to explore central subjects of content. The DISCERN instrument was used to assess the quality of information, and the LIDA tool was used to assess accessibility, usability, and reliability of the included websites.

**Results:**

The content on the majority of the websites included care and treatment of children with congenital heart defects (88%, 59/67), causes of congenital heart defects (88%, 59/67), symptoms of congenital heart defects (85%, 57/67), prevalence of congenital heart defects (81%, 54/67), potential complications of congenital heart defects (75%, 50/67), prenatal diagnostics/screening methods (72%, 48/67), and specific congenital heart defects (72%, 48/67), whereas less than 10% included information about termination of pregnancy (6%, 4/67), care during pregnancy (5%, 3/67), and information specifically directed to partners (1%, 1/67). The mean of the total DISCERN score was 27.9 (SD 9.7, range 16-53). According to the instrument, a majority of the websites were categorized as very poor regarding information about effects of no treatment (88%, 59/67), support for shared decision making (85%, 57/67), achievement of its aims (84%, 56/67), explicit aims (82%, 55/67), risks of each treatment (82%, 55/67), how treatment choices affect overall quality of life (76%, 51/67), and areas of uncertainty (76%, 51/67). The mean of the total LIDA score was 92.3 (SD 13.1, range 61-127). According to the tool, a majority of the websites were categorized as good with regard to registration (97%, 65/67) and browser test (75%, 50/67), whereas a majority were categorized as poor with regard to currency (87%, 58/67), content production (84%, 56/67), and engagability (75%, 50/67).

**Conclusions:**

Difficulties in finding relevant information sources using Web search engines and quality deficits on websites are an incentive for health professionals to take an active part in providing adequate and reliable information online about congenital heart defects.

## Introduction

Globally, an increasing number of health care consumers use the Internet to search for health-related issues [1-4]. The Internet has the potential to provide highly accessible, interactive, and tailored information. However, this might be limited by navigational difficulties and inaccurate or misleading information that has not been peer-reviewed [5,6]. Although many individuals have little or no trust in Internet information, it is used as a primary source when searching for health-related information [7]. Studies of literacy practices in relation to health communication have shown that trustworthiness is the key issue for patients assessing health information. For example, pregnant women in the United Kingdom were shown to search for texts written by medical professionals or published by medical institutions, thus appraising authority on the basis of their trust in academic and professional expertise [4].

Advances in prenatal screening have improved the detection rate of fetal diagnoses of congenital heart defects (CHD) [8,9]. Following diagnosis, counseling from health professionals is essential regarding a wide range of topics, including, for example, the nature and consequences of the CHD, severity, treatments available, prognosis, postoperative complications, and possible associations of CHD with other diseases [10,11]. Based on the information received, the pregnant woman also has the option of and the responsibility for deciding whether or not to terminate the pregnancy. Depending on national legislation on termination of the pregnancy, the decision must often be made soon after receiving the diagnosis. The process towards an informed decision on the future of the pregnancy involves various difficulties, including comprehending complex medical information [12,13], ethical considerations [14,15] and psychological distress [16]. An online survey among parents of children with CHD revealed that 50% report that more information at the time of diagnosis would have been helpful [17]. To deal with this matter, pregnant women and their partners try to supplement counseling from health professionals by using the Internet to search for information following the diagnosis [12].

The aim of this study was to explore central subjects of content and to assess the accessibility, usability, reliability, and quality of written information on publicly available information websites about congenital heart defects following a prenatal diagnosis.

## Methods

### Data Collection

**Figure 1:**
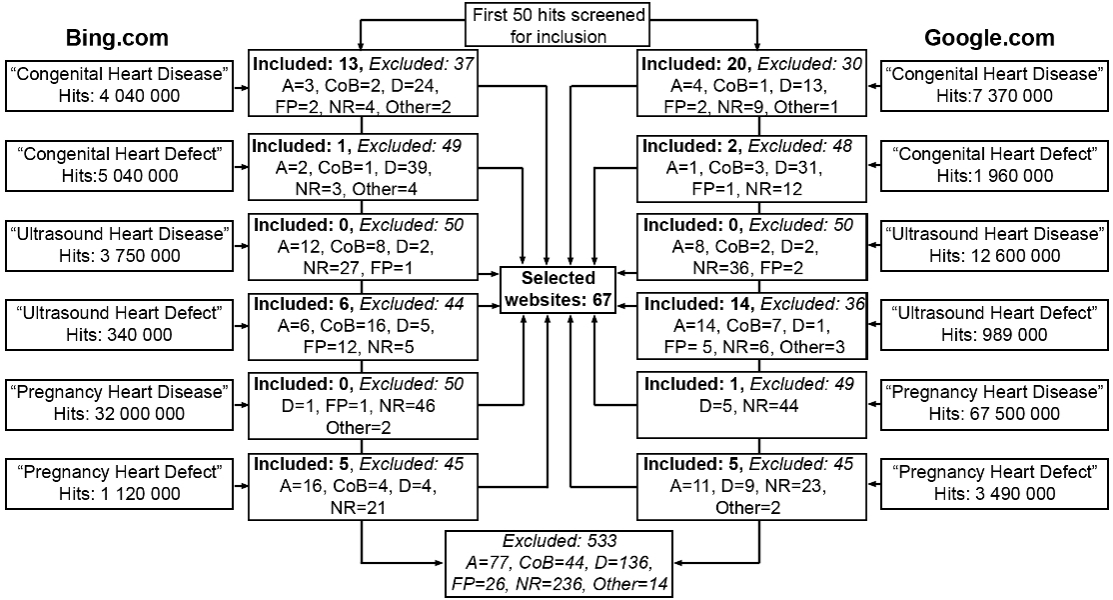
Selection process for the included websites (N=67; A: Scientific Article; CoB: Community or Blog; D: Duplicate; FP: For Professionals; NR: Not Relevant).

In October 2013, the following key terms were entered separately in the two most commonly used search engines, Bing and Google [18]: “Congenital Heart Disease”, “Congenital Heart Defect”, “Ultrasound Heart Disease”, “Ultrasound Heart Defect”, “Pregnancy Heart Disease”, and “Pregnancy Heart Defect”. The inclusion criterion was a website written in English that provided patient information regarding CHD. The search was made in incognito mode in order to minimize influence from previous search patterns. The generated result of the search procedure was saved, and the first 50 hits obtained for each search procedure were screened for inclusion (N=600). Duplicate websites and direct links to communities/blogs, video materials, and scientific articles were excluded. In total, 533 (88.8%) were excluded, leaving 67 (11.2%) websites for inclusion in the study. Figure 1 presents the selection process, that is, key terms, hits, and excluded and included websites via Bing and Google, respectively.

The websites we included were affiliated with independent information websites (25/67, 37%), charity/private organizations (17/67, 25%), hospitals/clinics (17/67, 25%), governments (4/67, 6%), medical companies (1/67, 2%), and other websites (4/67, 6%). The majority of the websites could not be attributed to an author (51/67, 76%), whereas a minority could be attributed to medical professionals (10/67, 15%), journalists (2/67, 3%), and others (4/67, 6%).

### Data Analyses

#### Central Subjects of Content

A manifest content analysis [19] was performed to inductively identify the central subjects of content of each website. The first author read the websites repeatedly. Paragraphs and statements (meaning units) containing relevant information regarding the content of each website were identified and grouped into categories (central subjects). Meaning units in the same category are assumed to have a similar meaning, on the basis of either the precise meaning of text or of texts sharing the same connotations. Thereafter, the websites were read again and subcategories were identified, that is, common characteristics within a larger category. Finally, the websites were all read through once more in order to validate the results.

#### Assessment of Quality

The websites were individually evaluated using two standardized instruments: the DISCERN instrument [20] and the Minervation validation instrument for health care websites (LIDA tool, version 1.2) [21]. The first author conducted all assessments of the websites.

The DISCERN instrument is a reliable and valid instrument for assessing the quality of written consumer health information independent of previous knowledge of the field under research [20]. It was developed with the input of an expert panel, health information providers, and patients from a self-help group, and has acceptable levels of interrater agreement [22]. The instrument consists of 16 questions divided into three sections. The user rates each question on a scale ranging from 1 (low/poor) to 5 (high/excellent), resulting in a total score ranging from 16 to 80. Section 1 includes 8 questions (score ranging from 8-40) and assesses reliability, whereas Section 2 with 7 questions (7-35) focuses on the quality of information about treatment options, that is, in this study continuation/termination of pregnancy, fetal interventions, and treatments of CHD. Section 3 consists of 1 question (1-5) and provides an overall rating of the quality of the websites, based on the responses to the previous questions [20].

The LIDA tool [21] assesses accessibility, reliability, and usability of health care websites. It consists of 29 questions and an automated test. Each question is rated from 0 to 3 (higher being better), and the automated test generates a score of 0-54, resulting in a total score ranging from 0 to 141. The overall score is calculated as a percentage, where scores greater than 90% represent good results and less than 50% poor results [23]. Accessibility (score ranging from 0-60) includes an automated test of page set-up, access restrictions, and outdated code, together with manual registration and browser tests conducted in Apple Safari, Google Chrome, and Mozilla Firefox. Usability (0-54) includes clarity, consistency, functionality, and engagability. Reliability (0-27) includes currency, conflicts of interest, and content production.

#### Statistical Analysis

Descriptive statistics were carried out using R (version 3.0.1).

## Results

### Central Subjects of Content

Central subjects of content on the 67 websites were categorized into 25 categories with 46 subcategories (Table 1).

**Table 1:** Central subjects of content identified on the included websites (N=67).

Category	Subcategory	n (%)
**Care and treatment of children with CHD**		59 (88)
	Surgery	59 (88)
	Cardiac catheterization for treatment	48 (72)
	Medications	48 (72)
	Cardiac transplantation	39 (58)
	Nutrition	18 (27)
	Pacemaker	17 (25)
	Intensive care	11 (16)
	Animations or illustrations of treatments	9 (13)
	Immunizations	3 (5)
	How to include cultural/spiritual beliefs in the care of the child	1 (1)
Causes of CHD		59 (88)
Symptoms of CHD		57 (85)
Prevalence of CHD		54 (81)
Potential complications of CHD		50 (75)
**Prenatal diagnostic/screening methods**		48 (72)
	Fetal echocardiography	40 (60)
	Amnioscentesis	15 (22)
	Chorionic villus sampling	12 (18)
	Nuchal translucency scan	11 (16)
	Blood tests	9 (13)
	Risks of invasive methods	9 (13)
	Umbilical cord sampling	4 (6)
	Fetal magnetic resonance imaging	2 (3)
**Specific CHD**		48 (72)
	Animations or illustrations of CHD	28 (42)
Associated anomalies		47 (70)
**Normal cardiovascular system**		46 (69)
	Postnatal cardiovascular system	42 (63)
	Cardiovascular changes at birth	35 (52)
	Animations or illustrations of normal cardiovascular system	29 (43)
	Fetal cardiovascular system	16 (24)
**Postnatal diagnostic methods**		45 (67)
	Echocardiography	43 (64)
	Electrocardiography	42 (63)
	Physical examination	42 (63)
	Radiography	42 (63)
	Cardiac catheterization for diagnosis	39 (58)
	Pulse oximeter	25 (37)
	Chemical analyses	18 (27)
	Exercise test	16 (24)
**Long-term outlook and care**		44 (66)
	Monitoring/Follow-up care	31 (46)
	Dental care/endocarditis prophylaxis	28 (42)
	Grown-up with CHD	24 (36)
	Pregnancy with CHD in mother	24 (36)
	Physical activity	23 (34)
Prognosis		41 (61)
Risks of treatment of CHD		25 (37)
Common feelings following prenatal diagnosis of CHD		18 (27)
**Postnatal quality of life**		18 (27)
	Quality of life for the child	18 (27)
	Quality of life for the family	5 (7)
Examples of previous cases that continued the pregnancy		16 (24)
Precision of prenatal diagnosis of CHD		16 (24)
**Delivery**		14 (21)
	Location and planning of delivery	14 (21)
	Mode of delivery	1 (1)
**Postnatal coping with the diagnosis**		13 (19)
	Financial issues	12 (15)
	Grief and bereavement	2 (3)
	Information regarding siblings	2 (3)
Risks of CHD in future pregnancy		10 (15)
Fetal intervention		8 (12)
Presentation of the multidisciplinary team in care of the child		8 (12)
**Termination of pregnancy**		4 (6)
	Informed and personal decision	2 (3)
	Time limit	2 (3)
	Feelings about termination of pregnancy	1 (1)
Care during pregnancy		3 (5)
Information specifically directed to partners		1 (1)

The majority (>70%) of the websites contained information about care and treatment of children with CHD (88%, 59/67), causes of CHD (88%, 59/67), symptoms of CHD (85%, 57/67), prevalence of CHD (81%, 54/67), potential complications of CHD (75%, 50/67), prenatal diagnostics/screening methods (72%, 48/67), and specific CHD (72%, 48/67). A minority (<30%) of the websites contained information about common feelings following prenatal diagnosis of CHD (27%, 18/67), postnatal quality of life (27%, 18/67), examples of previous cases that continued the pregnancy (24%, 16/67), precision of prenatal diagnosis of CHD (24%, 16/67), delivery (21%, 14/67), postnatal coping with the diagnosis (19%, 13/67), risks of CHD in future pregnancy (15%, 10/67), fetal intervention (12%, 8/67), presentation of the multidisciplinary team in care of the child (12%, 8/67), termination of pregnancy (6%, 4/67), care during pregnancy (5%, 3/67), and information specifically directed to partners (1%, 1/67).

### Assessment of Quality

#### DISCERN

Table 2 presents means, standard deviations, and ranges of the included websites measured by the DISCERN instrument.

**Figure 2:**
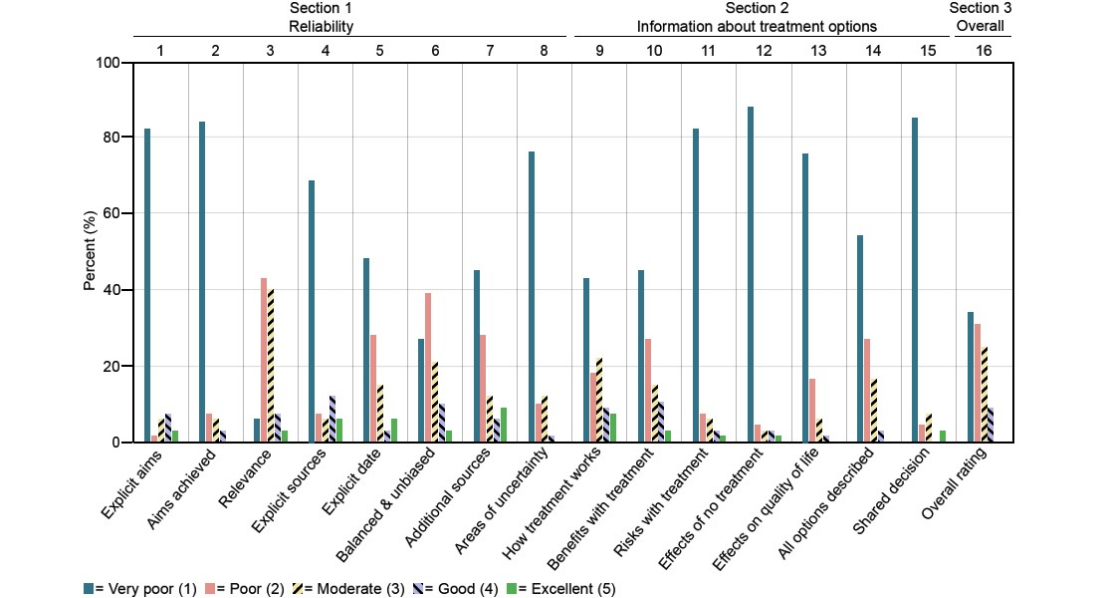
Proportion of websites (N=67) categorized into five categories from very poor to excellent, for each separate question in the DISCERN instrument.

In applying the DISCERN criteria to the evaluation of the websites (Figure 2), the majority of the websites (>70%) were categorized as very poor regarding effects of no treatment (88%, 59/67), support for shared decision making (85%, 57/67), achievement of its aims (84%, 56/67), explicit aims (82%, 55/67), risks of each treatment (82%, 55/67), how treatment choices affect overall quality of life (76%, 51/67), and areas of uncertainty (76%, 51/67).

**Table 2:** Means, standard deviations (SD), and ranges of the included websites (N=67) measured by the DISCERN instrument (the maximum achievable scores in shown in brackets after each section and question).

Section (max. score)	Question	Mean (SD)	Range
**Reliability (40)**		14.7 (5.2)	8-30
	Explicit aims (5)	1.5 (1.1)	1-5
	Aims achieved (5)	1.3 (0.7)	1-4
	Relevance (5)	2.6 (0.8)	1-5
	Explicit sources (5)	1.8 (1.3)	1-5
	Explicit date (5)	1.9 (1.1)	1-5
	Balanced and unbiased (5)	2.2 (1.1)	1-5
	Additional sources(5)	2.1 (1.3)	1-5
	Areas of uncertainty (5)	1.4 (0.8)	1-4
**Treatment options (35)**		11.1 (4.9)	7-25
	How treatment works (5)	2.2 (1.3)	1-5
	Benefits of treatment (5)	2.0 (1.1)	1-5
	Risks of treatment (5)	1.3 (0.8)	1-5
	Effects of no treatment (5)	1.3 (0.8)	1-5
	Effects on quality of life (5)	1.3 (0.7)	1-4
	All options described (5)	1.7 (0.9)	1-4
	Shared decision (5)	1.3 (0.9)	1-5
Overall rating (5)		2.1 (1.0)	1-4
Total (80)		27.9 (9.7)	16-53

#### LIDA

Table 3 presents means, standard deviations (SD), and ranges of the included websites measured by the LIDA tool.

**Figure 3:**
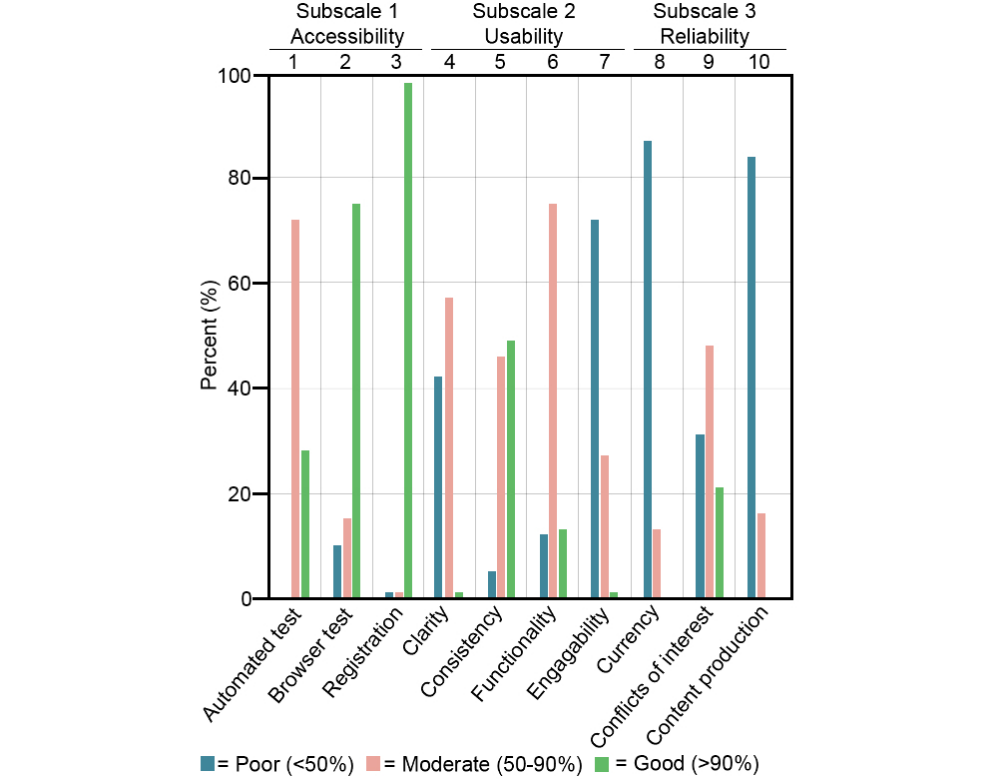
Proportion of websites (N=67) categorized into three categories from poor to good, for the subscales and total LIDA score.

In applying the LIDA criteria to the evaluation of the websites (Figure 3), the majority of the websites (>70%) were categorized as good regarding registration (97%, 65/67) and browser test (75%, 50/67), whereas the majority was categorized as poor regarding currency (87%, 58/67), content production (84%, 56/67), and engagability (75%, 50/67).

**Table 3:** Means, standard deviations, and ranges of the included websites measured by the LIDA tool (the maximum achievable scores in shown in brackets after each section and question).

Section (max. score)	Subscale	Mean (SD)	Range
**Accessibility (60)**		50.7 (5.3)	37-59
	Automated test (54)	45.1 (5.4)	31-53
	Browser test (3)	2.6 (0.7)	1-3
	Registration (3)	3.0 (0.3)	1-3
**Usability (54)**		32.5 (7.1)	19-48
	Clarity (18)	9.8 (3.2)	3-17
	Consistency (9)	7.9 (1.5)	3-9
	Functionality (15)	10.6 (2.4)	6-15
	Engagability (12)	4.2 (2.6)	1-11
**Reliability (27)**		8.9 (5.4)	0-22
	Currency (9)	2.0 (1.8)	0-7
	Conflicts of interest (9)	5.6 (2.9)	0-9
	Content production (9)	1.5 (2.2)	0-7
Total (141)		92.3 (13.1)	61-127

## Discussion

### Principal Results

We searched the Internet with different key terms to find publicly available patient information following a prenatal diagnosis of a congenital heart defect. Hits ranged from 350,000 to 67,500,000 and 67 of 600 screened websites were included in the study. Over a third (37%, 25/67) of the websites were affiliated with independent information sources, whereas a quarter (25%, 17/67) were affiliated with hospitals/clinics. The majority of the information on the websites (76%, 51/67) could not be attributed to an author. A minority of the websites contained information regarding certain prenatal aspects, that is, common feelings following prenatal diagnosis of CHD, precision of prenatal diagnosis, delivery, risks of CHD in future pregnancy, fetal intervention, termination of pregnancy, and care during pregnancy. Furthermore, the majority of the included websites were scored very poor by the DISCERN instrument with regard to information about effects of no treatment, support for shared decision making, achievement of its aims, explicit aims, risks of each treatment, how treatment choices affect overall quality of life, and areas of uncertainty. The reliability of the included websites was poor according to both the DISCERN instrument and the LIDA tool, particularly regarding currency, content production, aims of website, and areas of uncertainty. However, the accessibility and usability of the included websites were sufficient.

The literature suggests that the overwhelming number of websites found when searching the Web for information leads to information overload and searching difficulties [5,12,24]. The fact that it is difficult to find relevant information on the Internet is exemplified in this study: 39.3% (236/600) of the screened websites were irrelevant. Thus, it is possible that persons seeking information about CHD miss accurate and valuable information or give up information retrieval via the Internet because of difficulties in finding relevant sources. This difficulty might be enhanced by the fact that cardiologists seldom give recommendations on websites in connection with the diagnosis [17]. Health care professionals need to be aware that parents of children with CHD rank information regarding websites at the time of diagnosis as more important than cardiologists [25]. Consequently, as health care consumers are increasingly using the Internet to search for information [1-4], health care professionals need to address these circumstances and actively strive to recommend and provide accurate and reliable high-quality information online.

It seems that the websites target families following a postnatal diagnosis or women opting to continue the pregnancy. Previous research suggests that induced abortion is viewed as a socially unacceptable and stigmatizing procedure [26], independent of state laws on pregnancy termination [27]. It could be speculated that this perspective influenced the content of the included websites, as few contained information regarding termination as an option following a prenatal diagnosis of CHD.

The Internet may provide inaccurate and biased material [5,28,29]. It is imperative that pregnant women make informed decisions regarding whether to continue or terminate the pregnancy, which may be hindered by unreliable information sources found online. The majority of the websites in this study had poor reliability in a number of areas, including currency, conflicts of interest, and content production. The importance of current and unbiased information is especially important in the context of the rapidly expanding and evolving field of fetal cardiology, in order to promote informed decisions.

Health literacy, that is, the degree to which individuals have the capacity to obtain, process, and understand the health information and services needed to make appropriate decisions [30], is an important concept when discussing disparities in health information comprehension. Illustrations as a complement to oral information can substantially increase comprehension of health information, are especially helpful for those with poor health literacy [31], and are desired in connection with initial diagnosis [12]. However, animations and illustrations were scarce among the reviewed Web pages. It is therefore possible that the information online is not suited for those with poor health literacy. Health care professionals need to acknowledge this and provide pedagogic tools to promote patient comprehension and equal care.

### Strengths and Limitations

This study did not evaluate the scientific quality of the reviewed websites, nor did it assess the accuracy of the information found, that is, if the included websites contained any inaccurate or misleading information. Furthermore, it is possible that the key terms do not fully represent the online landscape of websites about CHD and that other results would have emerged with different search methods. However, according to previous research, the majority of health information seekers use search engines as their primary source [1,32], and the search engines used in this study are at present the most commonly used [18]. Moreover, the searches yielded 136/600 (22.7%) duplicate websites, indicating saturation and that the searches do represent the online landscape.

The DISCERN instrument and LIDA tool are based on subjective ratings. Only the first author conducted the assessments, and this could imply poor generalizability, and perhaps also a certain bias. The DISCERN instrument, developed and designed to help users of consumer health information judge the quality of written information, has been found to be consistently understood as well as transferable to different specialties (eg, [20,23,33,34]). Furthermore, the first author is a nurse, which could possibly indicate different views than non-professionals and thus different scorings. However, previous research suggests that scorings are not dependent on previous knowledge of the specific condition [22], and it has been concluded that health professionals score DISCERN similarly to non-professionals when assessing health information [33]. Taken together, we find it reasonable to assume that the main outcomes from this study would have been similar even with another evaluator or with more than one evaluator.

Approximately 25% (17/67) of the websites were affiliated with hospital/clinics, and 76% (51/67) could not be attributed to an author. Consequently, it is important to bear in mind that the information found on the included websites may differ from the information provided by health care professionals following a diagnosis of CHD.

### Suggestions for Future Research

It remains unclear if websites about congenital heart defects following a prenatal diagnosis contain accurate and suitable information. This needs to be evaluated in future studies by health professionals within fetal/pediatric cardiology and persons with experience of a prenatal diagnosis of a CHD.

It has been reported that expectant parents want more information than that provided by health care professionals following a prenatal diagnosis of CHD [17]. The results from this study, however, indicate that existing websites do not adequately supplement counseling. Development of an information source via the Internet would enable expectant parents to access accurate and tailored information that complements the standard counseling offered today. In order to evaluate such tools, national, or even international, research collaborations are needed.

Easily accessible information on the Internet influences the conditions for doctor-patient interaction [35]. What can be communicated by the doctor, and acknowledged by the patient, always depends on the previous knowledge and perspective that the patient has developed in their own information seeking. Thus, more knowledge is needed on the communication chains in which the patients build their knowledge and understanding.

The linguistic readability of the websites was not assessed in this study. Neither was patients’ interpretation and evaluation of the information investigated. Several models for mechanical syntactic analysis, in order to measure readability, were developed in the early years of text linguistics, focusing mainly on factors such as word length and syntactic complexity [36,37]. Similar models have been used to assess medical information [23,34]. Later research in computational linguistics suggests that measures of semantics and discourse cohesion, that is, “content”, show higher correlations with reported readability [38]. To gain a deeper understanding of Web texts, high-quality qualitative text analytical studies are needed. Preferably, such studies should be combined with reader interviews, focusing on interpretation and comprehension. Furthermore, additional research is needed in order to understand how different types of illustrations can provide relevant understanding of the disease.

### Conclusions

The reviewed websites do not adequately supplement counseling from health care professionals following a prenatal diagnosis of CHD. Difficulties in finding relevant information sources using Web search engines and quality deficits on websites are an incentive for health professionals to take an active part in providing adequate and reliable information online about CHD. Future websites need to have a clearer prenatal perspective to become a source of knowledge for prospective parents seeking information online following diagnosis.

## Abbreviations

CHD: congenital heart defect
